# *Arabidopsis thaliana* Plant Natriuretic Peptide Active Domain Forms Amyloid-like Fibrils in a pH-Dependent Manner

**DOI:** 10.3390/plants11010009

**Published:** 2021-12-21

**Authors:** Georgia I. Nasi, Foteini D. Aktypi, Panagiotis M. Spatharas, Nikolaos N. Louros, Paraskevi L. Tsiolaki, Vassiliki Magafa, Ioannis P. Trougakos, Vassiliki A. Iconomidou

**Affiliations:** 1 Section of Cell Biology and Biophysics, Department of Biology, School of Sciences, National and Kapodistrian University of Athens, Panepistimiopolis, 157 01 Athens, Greece; gnasi@biol.uoa.gr (G.I.N.); faktipi@biol.uoa.gr (F.D.A.); panspatharas@biol.uoa.gr (P.M.S.); nlouros@biol.uoa.gr (N.N.L.); etsiolaki@biol.uoa.gr (P.L.T.); itrougakos@biol.uoa.gr (I.P.T.); 2 Department of Pharmacy, University of Patras, 265 04 Patras, Greece; magafa@upatras.gr

**Keywords:** *Arabidopsis thaliana*, amyloid fibrils, natriuretic peptides, plant natriuretic peptides, functional amyloid

## Abstract

Plant natriuretic peptides (PNPs) are hormones that have been extracted from many different species, with the *Arabidopsis thaliana* PNP (AtPNP-A) being the most studied among them. AtPNP-A is a signaling molecule that consists of 130 residues and is secreted into the apoplast, under conditions of biotic or abiotic stress. AtPNP-A has distant sequence homology with human ANP, a protein that forms amyloid fibrils in vivo. In this work, we investigated the amyloidogenic properties of a 34-residue-long peptide, located within the AtPNP-A sequence, in three different pH conditions, using transmission electron microscopy, X-ray fiber diffraction, ATR FT-IR spectroscopy, Congo red and Thioflavin T staining assays. We also utilize bioinformatics tools to study its association with known plant amyloidogenic proteins and other *A. thaliana* proteins. Our results reveal a new case of a pH-dependent amyloid forming peptide in *A. thaliana*, with a potential functional role.

## 1. Introduction

Amyloid fibrils are formed by proteins or peptides, that under certain conditions self-assemble into characteristic fibrillar structures [1]. These highly ordered structures are characterized by extreme stability, while conflicting evidence has emerged about the ability of proteases to fragment them [2,3]. Amyloid forming proteins do not share any similarity in sequence or native structure, albeit amyloid fibrils are characterized by a common structure, known as “cross-β” structure. In “cross-β” conformation, hydrogen-bonded β-strands are organized perpendicularly to the fibril axis shaping β-sheets, which are, in turn, organized parallel to the main axis of the fibril [4]. Amyloid fibrils can be characterized either as pathological or functional; the deposition of amyloid fibrils is the main hallmark of a group of conformational disorders, known as amyloidoses [5], while on the other hand, many organisms, ranging from bacteria to humans, exploit the properties of amyloid fibrils to perform physiological functions [6,7].

In humans, the major hallmark of isolated atrial amyloidosis (IAA) is the formation of fibrillar deposits in the atria of the aging heart. Their primary component is the atrial natriuretic peptide (ANP) [8], a small hormone that belongs to the family of natriuretic peptides (NPs) and consists of 28 amino acid residues [9]. Nevertheless, immunohistochemical studies have shown that other NPs are also present on these deposits, such as brain NP (BNP) [10] and proANP_1–98_ [11]. ANP regulates blood volume and pressure in the circulatory system, via binding to a cell surface receptor, namely the natriuretic peptide receptor-A (NPR-A) [12,13]. In 1991, Vesely and Giordano also discovered the existence of the NPs system in plants, after using antibodies against human ANP (hANP) in plant tissue extracts [14]. Studies supported this groundbreaking finding, and further expanded on the role of plant natriuretic peptides (PNPs) [15,16,17,18] suggesting that they actively contribute to protoplast cell volume regulation [19,20,21] and to the ion balance of plants [22]. Impressively, PNPs act upon binding to cell membrane receptors in the same fashion as hANP indicating that they activate similar signaling pathways [23,24,25].

**Figure 1:**
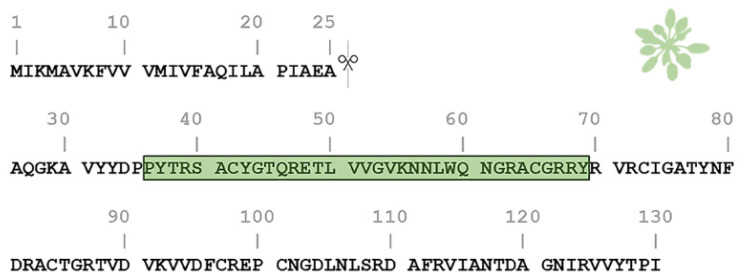
Amino acid sequence of the *Arabidopsis thaliana* plant natriuretic peptide (AtPNP-A). The pre-processed sequence of AtPNP-A (UniProt AC: Q9ZV52) consists of 130 residues and contains a signal peptide in its N-terminal (residues 1–25), responsible for its secretion. The active domain of the AtPNP-A molecule (AtPNP-A_36–69_) corresponds to the 36–69 region (green box).

One of the most studied members of the PNP family is the *Arabidopsis thaliana* PNP (AtPNP-A). AtPNP-A is a signaling molecule that is secreted into the apoplast under conditions of biotic and abiotic stress in order to regulate cell response, thus preserving plant homeostasis [26,27,28]. The pre-processed sequence of AtPNP-A consists of 130 residues and contains a 25-residue-long N-terminal signal peptide which is critical for its secretion into the apoplast [28,29]. The AtPNP-A sequence contains a 34 amino acid region (AtPNP-A_36–69_), which is pivotal for its biological activity [21] (Figure 1). This peptide was also found to be conserved among all PNPs [20].

In the early 2000s, Ludidi et al. suggested that the hANP displays a distant sequence homology (ca. 30%) with the 34aa active domain of AtPNP-A and presented their alignment, based on their sequence similarity (Figure S1) [20,29]. The two proteins have a similar course of action after secretion, as they bind to membrane receptors and activate similar signaling pathways involved in ion balance and water movement regulation [13,22]. Additionally, it is known that all vertebrate NPs are conserved and share a common structure of a 17aa ring formed by a disulfide bond, that has been proved to be essential for their biological activity [30]. Experiments that followed, showed that animal NPs can induce responses in plants similar to PNPs and vice versa [31,32], while Gehring et al. provided evidence that the NPs must maintain their 17aa loop structure in order to exhibit this biological activity in plants [33]. Especially in the case of hANP and AtPNP-A, it has been shown that they induce similar functional effects in plants, while AtPNP-A can affect cardiomyoblast cell lines, inducing apoptosis similarly to hANP [32,34,35,36]. When it comes to other PNPs, multiple alignments of all the recently available PNPs from several plant species (Figure S2) show relative conservation in the sequences that correspond to the 34aa domain of AtPNP-A. It is noteworthy that all the PNP sequences include a pair of cysteine residues, a fact which indicates that the loop ring structure is presumably present and essential for the biological activity of PNPs.

Taking into consideration the well-established amyloidogenicity of hANP [11,37], along with the functional similarity to its plant homologue, we decided to investigate the aggregation properties of the homologous region that corresponds to the biologically active and conserved functional domain of AtPNP-A [32,33] (Figure S1). Using biophysical methods, we demonstrate that this region (amino acid residues 36–69) of AtPNP-A self-assembles into fibrils with characteristic amyloid properties in three different pH conditions. We also provide computational evidence for the potential implication of *A.thaliana* proteins—associated with AtPNP-A—in amyloid fibril formation. Our data add to the heterogeneous list of proteins shown to form amyloids in plants and is expected to promote other computational and experimental studies on plants in the field of amyloid biology.

## 2. Results

The ability of AtPNP-A_36–69_ to self-assemble and form fibrils with amyloidogenic properties was tested in three different pH conditions (pH 5.6, 7.4 and 8.4), and each solution was incubated for 2–3 weeks at 37 °C temperature. The characterization of its aggregates as amyloid-like fibrils was based on the observation of their morphological and structural characteristics according to the basic criteria for the identification of amyloid fibrils [38,39].

### 2.1. AtPNP-A_36–69_ Forms Amyloid-like Fibrils under Three Different pH Conditions

**Figure 2:**
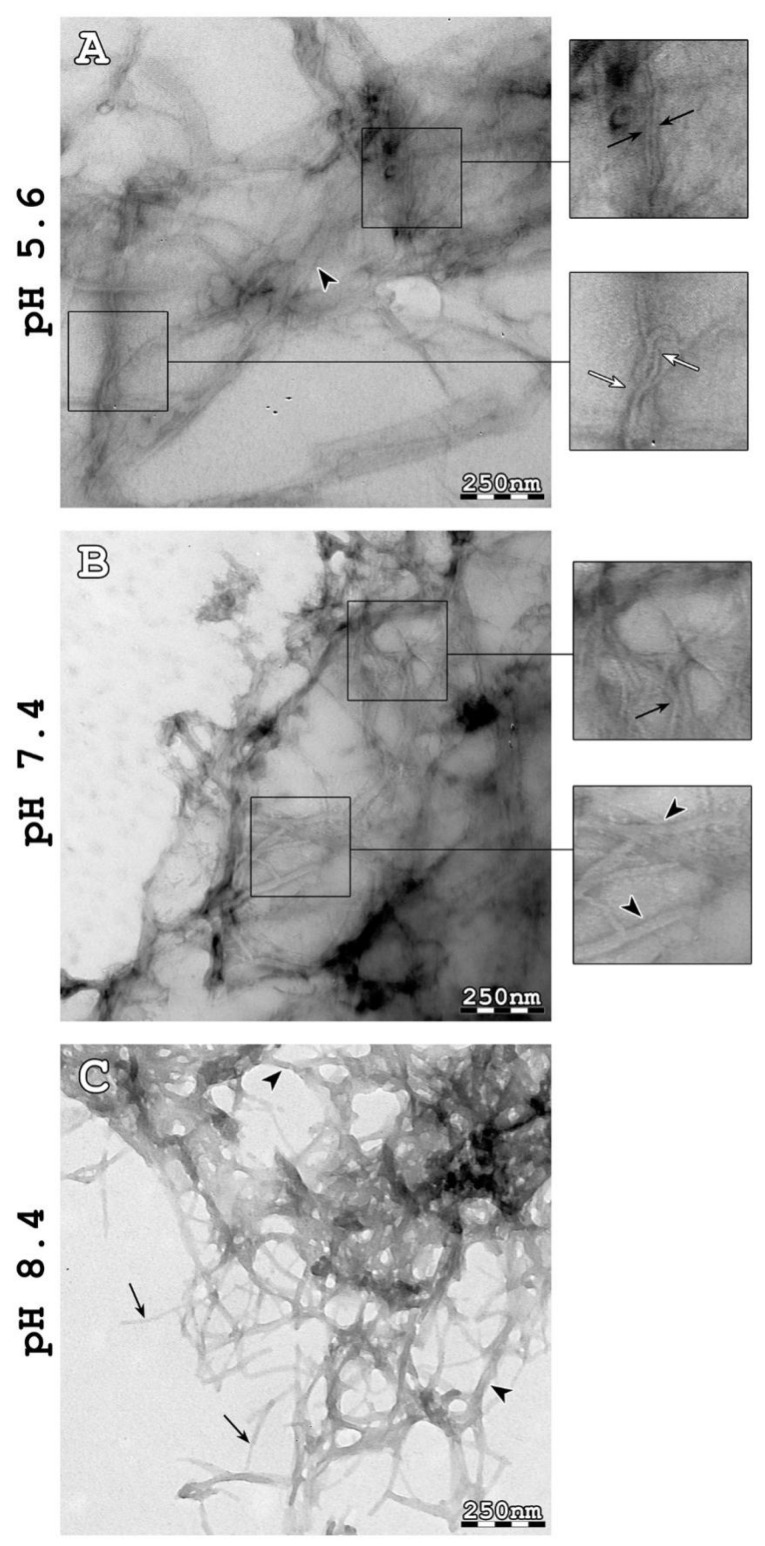
Transmission electron micrographs of amyloid-like fibrils derived from AtPNP-A_36–69_ in three different pH conditions. (**A**) When incubated at pH 5.6 (MES buffer), the thinnest AtPNP-A_36–69_ fibrils had a diameter of approximately 70 Å (black arrows). These fibrils interacted, forming either ribbon-like (black arrowheads) or twisted (white arrows) structures. (**B**) When incubated at pH 7.4 (HEPES buffer), AtPNP-A_36–69_ formed amyloid fibrils, with a diameter of approximately 70 Å (black arrows). The fibrils formed ribbon-like structures with various widths (black arrowheads). (**C**) When AtPNP-A is incubated at pH 8.4 (Tris buffer), it formed straight, unbranched and of undefined length fibrils, with a diameter ranging from approximately 90 Å to 100 Å (black arrows). In this case, ribbon-like structures were also observed (black arrowheads). Bar 250 nm.

To investigate our hypothesis, that AtPNP-A_36–69_ forms amyloid fibrils, as well as the effect of each pH value on them, we inspected the morphology of the aggregates formed in each condition using Transmission Electron Microscopy (TEM) after negative staining (uranyl acetate 2% (*w*/*v*)). To achieve this, a sample from each of the three tested solutions was taken after an incubation period of 2 weeks at 37 °C temperature. The concentration of all peptide solutions was 20 µmol/L. Transmission electron micrographs showed that the aggregation of AtPNP-A_36–69_ led to a considerable variety of structural polymorphs depending on the pH conditions. When incubated at pH 5.6 (MES buffer), the thinnest fibrils had a diameter of approximately 70 Å and underwent further self-assembly forming both ribbon-like and twisted structures of various widths (150–300 Å; Figure 2A—black arrowheads and white arrows, respectively). Moreover, non-ordered structures were detected. When incubated at pH 7.4 (HEPES buffer), the thinnest fibrils observed had approximately the same diameter as MES-prepared individual fibrils. However, the fibrils tended to only form ribbons (150–300 Å; Figure 2B—black arrowheads) and not twisted structures. Finally, when incubated at pH 8.4 (Tris buffer), AtPNP-A_36–69_ formed straight and unbranched fibrils with an undefined length (several μm long) (Figure 2C), features consistent with those appearing in other amyloidogenic material. The thinnest fibrils had a diameter of approximately 90–100 Å and were packed more densely when compared to the ones formed at lower pH conditions. The ribbon-like structures were also preserved in this pH level (Figure 2C—black arrowheads).

### 2.2. Characterization of the Structure of AtPNP-A_36–69_ Amyloid-like Fibrils

**Figure 3:**
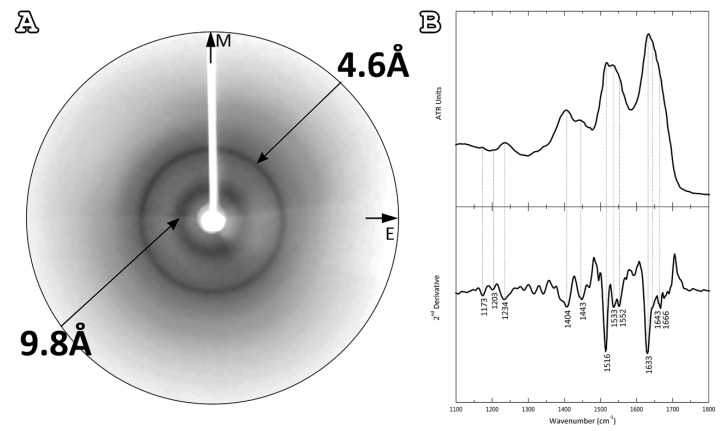
Structural features of AtPNP-A_36–69_ fibrils. (**A**) X-ray diffraction pattern produced by oriented fibers containing AtPNP-A_36–69_ amyloid fibrils. The pattern is indicative of the “cross-β” structure, displaying both a meridional (M) 4.6 Å and an equatorial (E) 9.8 Å reflection, corresponding to the distance between consecutive β-strands and the distance between packed β-sheets, respectively. The random packing of the fibrils results in the occurrence of rings instead of oriented reflections. (**B**) ATR FT-IR (1100–1800 cm^−1^) spectra results, obtained from suspensions of fibrils, produced from the AtPNP-A_36–69_, cast on a flat stainless-steel plate and left to air-dry slowly, at ambient conditions, to form hydrated, thin films. Second derivative spectra are also included. (see also Table 1).

Following amyloid-like fibril observation with electron micrographs, we wanted to examine their structure. To achieve this, an X-ray diffraction pattern was obtained from an oriented fiber placed on a capillary with a wax-covered end, formed by the 2.5 mmol/L aqueous solution. Similarly, with the solutions used during TEM observation, the sample was incubated for 2 weeks at 37 °C temperature. The derived X-ray diffraction pattern resembled the ‘‘cross-β’’ structure, usually found in other amyloidogenic proteins. Specifically, we observed a strong reflection at 4.6 Å that is attributed to the interchain distance between hydrogen-bonded β-strands and a less intense one at 9.8 Å, corresponding to the distance between packed β-sheets (Figure 3A). However, there was no preferential orientation of these two reflections along the meridian or the equator of the X-ray diffraction pattern, as we would expect in a typical “cross-β” pattern, implying a random packing of the constituent amyloid fibrils in the fiber.

As additional evidence, an attenuated total reflectance Fourier-transform infrared (ATR FT-IR) spectrum was obtained at a resolution of 4 cm^−1^ from a thin hydrated fibril-containing film, formed by the 2.5 mmol/L aqueous solution. ATR FT-IR spectral acquisitions indicated a predominant β-sheet secondary structure for the amyloid fibrils derived by the AtPNP-A_36–69_, supporting all previous observations (Figure 3B). More specifically, in the amide I region of the spectrum, a strong band at 1633 cm^−1^ was displayed, indicative of a β-sheet conformation. Another prominent band of particular interest, at 1666 cm^−1^, indicated the presence of β-turns, which most likely interconnect successive β-strands. Two equally significant bands confirmed the β-structure of our material: the first at 1552 cm^−1^ in the amide II region and the second at 1234 cm^−1^ in the amide III region. Finally, bands characteristic of TFA were observed, which was involved in the peptide synthesis and its remnants were detected, as well as the characteristic band of the aromatic residue tyrosine, that is found multiple times in the AtPNP-A_36–69_ sequence (Table 1).

### 2.3. Amyloidophilic Dye-Binding Assays

**Figure 4:**
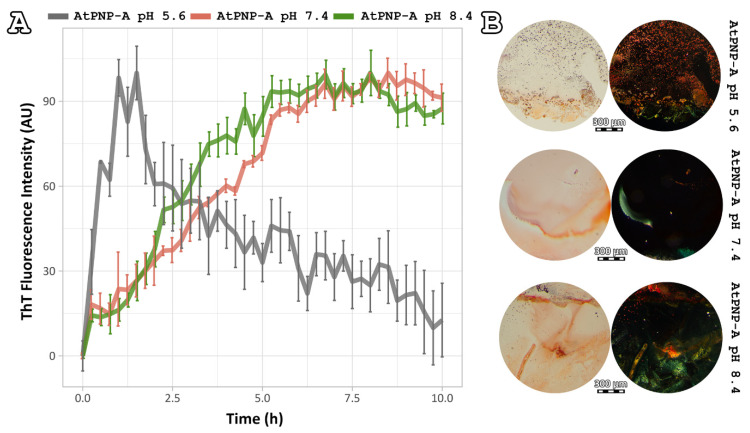
ThT and Congo Red assays of AtPNP-A_36–69_ amyloid-like fibrils. (**A**) ThT fluorescence emission spectrum of AtPNP-A_36–69_ incubated in three different pH conditions over a period of 10 h. AtPNP-A_36–69_, when dissolved in pH 7.4 (HEPES buffer) and 8.4 (Tris buffer), reached its peak after approximately 5 h (red and green line, respectively). When AtPNP-A_36–69_ is incubated in pH 5.6 (MES buffer), reached the highest value after approximately 1 h. Error bars in ThT fluorescence emission spectra represent standard deviation among triplicates. (**B**) Congo red staining results from the AtPNP-A_36–69_ fibrils. Photomicrographs of the fibrils (Left) under bright field illumination and (Right) under crossed polars. The typical for amyloid fibrils yellow/green birefringence clearly appears under crossed polars (Right). Bar 300 μm.

Thioflavin T (ThT) is known to specifically recognize and bind to amyloid fibrils. Upon binding, ThT exhibits a strong fluorescence signal at approximately 484 nm when excited at 444 nm [40], a property used to study the fibrillation kinetics of amyloid-forming peptides and proteins. To monitor the aggregation kinetics of AtPNP-A_36–69_ with ThT fluorescence, each of its mixtures was incubated at a concentration of 20 μmol/L at 37 °C for 50 h and ThT fluorescence measurements were collected every 15 min. AtPNP-A_36–69_, when dissolved at pH 8.4, reached its peak after approximately 5 h (Figure 4A—green line). The fibrillation process of AtPNP-A_36–69_, when incubated at pH 7.4, was slightly slower than that of the pH 8.4. (Figure 4A—red line). On the other hand, when AtPNP-A_36–69_ was incubated at pH 5.6, the lag phase was shorter, since the intensity reached the highest value after approximately 1 h.

It has been established that amyloid fibrils bind the Congo Red dye with high specificity and display a characteristic yellow/green birefringence when observed under crossed polars of a polarizing microscope [41]. Fibril-containing films of each peptide solution—after an incubation period of 2 weeks at 37 °C temperature—were stained with Congo Red and observed under polarizing stereomicroscope. Our results (Figure 4Β) indicated that the fibrils of the AtPNP-A_36–69_, in all three pH conditions, specifically bound the Congo Red dye, as shown by the sample exposure to bright field illumination (Figure 4Β, left). Under crossed polars (Figure 4Β, right), the characteristic yellow/green birefringence overlapped to Congo Red stained regions of the sample further confirmed the amyloid nature of the AtPNP-A_36–69_ fibrils. It is worth mentioning that the presence of yellow/green birefringence decreased as the pH value dropped.

## 3. Discussion

Our experimental results verify the fibrillar properties of the central domain of *Arabidopsis thaliana* PNP (AtPNP-A_36–69_), a distant homologue of hANP, as one of the few cases of amyloid-forming proteins in plants.

### 3.1. Implication of Different PH Values on AtPNP-A_36–69_ Fibril Formation

The aforementioned results suggest that plant amyloid fibrils have similar properties to the ones of mammalian origin, since in many cases the fibrillar assemblies of mammalian proteins differ depending on the incubation conditions, such as precursor monomer concentration [42] and pH [43,44,45]. Specifically, when it comes to pH changes, a-helix to β-strand conversion occurs in a variety of amyloid-forming proteins, such as Aβ peptide [46], β_2_-microglobulin [47], and transthyretin [48]. In most of these cases, amyloid formation is enhanced at slightly acidic pH values, while in basic pH values, these proteins form amorphous aggregates or a small amount of amyloids [49,50]. However, this work shows that AtPNP-A_36–69_ tends to form more robust and well-defined amyloid fibrils at higher pH values, than those at lower pH values, highlighting a difference between mammalian and plant proteins. AtPNP-A is a mobile molecule, mainly expressed under abiotic and biotic stress conditions [20], which favor the increase of pH of the apoplastic space of cells, as well as that of xylem sap [51,52]. These facts may imply a functional role of AtPNP-A when in an amyloid state, under stress and increasing pH values.

PNPs have a significant biotechnological interest regarding the genetic improvement of plants towards drought stress conditions and pathogen [22,53,54]. The AtPNP-A_36–69_ peptide constitutes the active domain throughout the PNP family and is a target for the genetic modification of the plant. The amyloidogenic properties of this sequence should be taken into consideration whenever we seek to overexpress this peptide [21].

### 3.2. Analysis of the AtPNP-A Network

**Figure 5:**
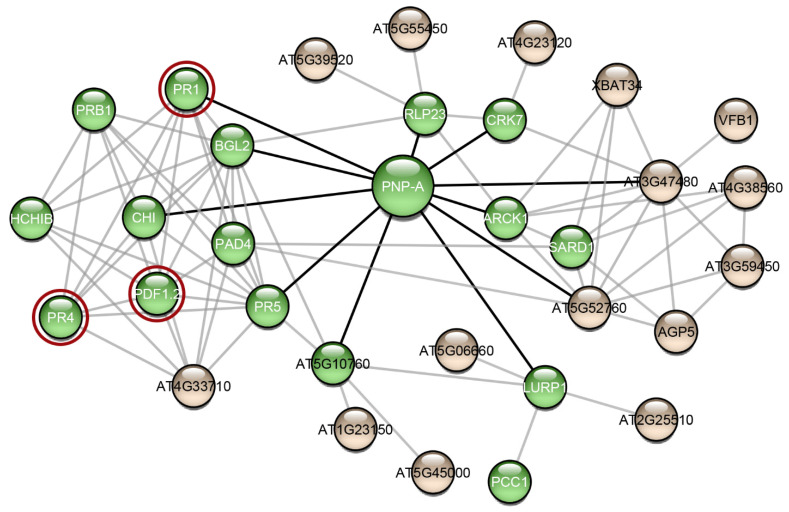
The interaction network of AtPNP-A. Interaction data for the creation of this network were gathered from the publicly available database STRING [57]. The network was visualized using Cytoscape 3.7.2 [58], a freely available platform for biological network visualization and analysis. The network consists of 32 nodes and 85 edges. Proteins are depicted as nodes and interactions as edges. Three of the proteins of the network (red circles) are associated with amyloid-forming proteins. Green-colored nodes are proteins that functional enrichment analysis indicated their association with defense responses. Black-colored edges are the interactions of AtPNP-A and grey-colored edges are the interactions of the AtPNP-A first neighbors.

Research interest has only recently started to focus on plant proteins with amyloidogenic properties. Computational analysis predicted that the *Arabidopsis thaliana* proteome appears to be abundant in proteins with amyloidogenic regions and a variety of different proteins were predicted as amyloidogenic candidates [55]. In this study, we wanted to find out if other proteins of *Arabidopsis thaliana* have been studied experimentally regarding their amyloidogenic properties, as well as their association with AtPNP-A, in order to unravel the importance of its ability to form amyloid fibrils. For this reason, we constructed the AtPNP-A network (Figure 5). From the 32 proteins of the network, only the pathogenesis-related protein-1 (PR1) has been studied regarding its amyloidogenic properties, showing that plant proteins are understudied regarding their amyloidogenic potential. Olrichs et al. tested the amyloidogenic potential of PR-1 under conditions which promote the amyloid formation of its respective mammalian counterpart [56]. As the authors point out, in the plant extracellular environment, PR-1 interacts with a completely different range of biomolecules or pathogens, and thus it is expected to not form amyloid fibrils under mammalian micro-environmental conditions, even though it contains “aggregation-prone” regions [56]. Our study of AtPNP-A produced similar results, supporting the conclusions of their study.

Another interesting observation that emerged from the study of the AtPNP-A network, is that most of the proteins in this network have been associated with functions related to defense responses (Figure 5—green spheres). Computational analysis showed that proteins with predicted “aggregation-prone” regions participate in defense responses, in the majority of plant species, implying that amyloid formation has a part in their function [55]. In fact, several plant defense proteins have been shown to have amyloid-like properties in vivo and in vitro. Vicilin, a seed storage protein, has been demonstrated to form amyloid fibrils both in vivo and in vitro. In particular, Antonets et al. showed that vicilin amyloids are present in pea seeds and its amyloid accumulation increased during seed maturation. Additionally, vicilin amyloids are toxic for fungi and bacteria, suggesting a functional dualism, being both a storage and a pathogen-defense protein [59]. Interestingly, vicilin consists of two β-barrel domains, cupin-1.1 and cupin-1.2, which also form amyloid fibrils in vitro. A homology model illustrating the native fold of AtPNP-A, as well as the model of the native state of *Xanthomonas axonopodis* PNP-like molecule, revealed that it may adopt a double-psi β-barrel structure, which is comprised of 6 β-strands, 2 α-helices, and 2 protruding psi loops [60]. According to this model, the active domain of AtPNP-A consists of two β-strands connected via an α-helix, which is in agreement with the results of the secondary structure prediction tool PORTER [61]. Moreover, taking a closer look at the secondary structure prediction of SECSTR [62] (Figure S3), AtPNP-A has regions with a propensity to form both α-helices and β-strands. Such sequences are characterized as “chameleon” sequences and, intrinsically, tend to alter their conformation depending on the environment [63]. Further studies regarding the native structure of AtPNP-A are needed to gain insight into its different roles and in order to unravel whether its β-barrel structure plays a role to the formation of amyloid.

RsAFP-19, a segment of the *Raphanus sativus* antifungal protein 1 and 2 [64], Cn-AMP2 of *Cocos nucifera* [65], and pro-hevein from *Hevea brasiliensis* [66], all have antimicrobial or antifungal properties while being amyloidogenic. Regarding the AtPNP-A network proteins, defensin-like protein 16 (Figure 5—PDF1.2) has high sequence similarity with the *Raphanus sativus* antifungal protein 1 (96%) and 2 (90%), suggesting that PDF1.2 likely possesses the same amyloidogenic properties as these proteins (Figure S4). Additionally, hevein-like pre-pro-protein (Figure 5—PR4), which is also found in the AtPNP-A network, has approximately 50% identity with pro-hevein (Figure S4). Therefore, it is possible that the respective *A. thaliana* defense proteins also form amyloid fibrils, a property which may be related to their function.

Specifically, it is believed that AtPNP-A is implicated in defense mechanisms as well. It has been shown that the transcriptional activation of AtPNP-A is triggered after a pathogen invasion, such as *Agrobacterium tumefaciens* [54]. Moreover, AtPNP-A is co-expressed with the Systemic Acquired Resistance marker genes—genes associated with defense against pathogens—and triggers the expression of many other genes important for plant defense [54,67]. These observations led Meier et al. to suggest the classification of AtPNP-A as a PR protein [54]. Consequently, the amyloidogenic properties of AtPNP-A may play a central role in the critical defense response mechanism of the plant, which remains to be uncovered.

## 4. Materials and Methods

### 4.1. Peptide Synthesis and Purification

A peptide-analogue corresponding to the 36–69 region of AtPNP-A (AtPNP-A_36–69_), homologous to the circulating form of the hANP peptide, was chemically synthesized. All 9-Fluorenylmethoxycarbonyl (Fmoc)-protected amino acids and 2-chlorotrityl-chloride resin were obtained from CBL Patras. Peptide reagents were purchased from (Bachem AG, Bubendorf, Switzerland). All solvents and reagents used for solid-phase synthesis were of analytical quality and used without further purification.

#### 4.1.1. Solid-Phase Synthesis of Linear Peptide

The linear precursor peptide-analogue was synthesized by Fmoc solid-phase methodology [68], utilizing a 2-chlorotrityl-chloride resin [69] as solid support to provide peptide with C-terminal carboxylic acid. Fmoc-protected amino acids were used with the trityl group (Trt) [Asn, Gln], the tert-Butyl group (But) [Tyr, Thr, Ser, Cys], the 2,2,4,6,7-pentamethyldihydrobenzofuran-5-sulfonyl group (Pbf) [Arg], and tert-butyloxy-carbonyl (Boc) [Trp, Lys] as side-chain protection groups. Stepwise synthesis of the peptide-analogue was achieved with Diisopropylcarbodiimide/Hydroxybenzotriazol (DIC/HOBt) as coupling agents in dimethylformamide (DMF) [70,71] in 3 (Fmoc-amino acid), 3.3 (DIC), and 4.5 (HOBt) molar excess for 2 h at room temperature. The completeness of the reaction was monitored by the Kaiser test [72]. Amino acid couplings were repeated if incomplete coupling was observed. Fmoc-deprotection was performed using treatments with 20% (*v*/*v*) piperidine-DMF for 5, 15, 10 min respectively. Complete Fmoc-cleavage was checked by running TLC after releasing the derivative from an aliquot of resin with the cleavage mixture AcOH–TFE–CH2Cl2 (1:2:7, *v*/*v*). After the removal of the last Fmoc-group from the N-terminal, the peptide was cleaved from the resin with trifluoroacetic acid/1,2-ethanedithiol/anisole/triethyl silane/water (92:3:2:2:1, *v*/*v*) (15 mL/g peptide resin) for 1 h at 0 °C and for 3 h at room temperature. All protecting groups, except But from cysteine residues, were removed under this condition. The resin was filtered off, the solvent was removed on a rotary evaporator and the product was precipitated as a white solid by the addition of cold and dry diethyl ether. The crude peptide with But protecting groups at Cys7,30 was used at the next step without purification. The disulfide bond is formed in one step simultaneously by the removal of But protecting groups using the silyl chloride-sulfoxide system for disulfide bond formation of cysteine peptide [73,74].

#### 4.1.2. Formation of Disulfide Bridge

The linear peptide with But protecting groups at Cys7,30 was dissolved in trifluoroacetic acid (TFA) at a peptide concentration of 1 µmol/mL, then treated with CH_3_SiCl_3_ (150 equiv) and PhS (O) Ph (10 equiv) mixture at RT for 20 min for development of disulfide bond. The product was precipitated by adding dry ether and the solid material was separated by centrifugation.

#### 4.1.3. Purification and Analysis by RP-HPLC

The crude disulfide peptide was initially purified by gel filtration chromatography on Sephadex G-25 using 15% acetic acid as the eluent. Final purification was achieved by semipreparative high-performance liquid chromatography (HPLC) (Mod. 10 ÄKTA, Amersham Biosciences, Piscataway, NJ, USA) on Supelcosil C18 (5 μm particle size, 25 cm × 8 mm, Sigma-Aldrich, St Louis, MO, USA) with a linear gradient elution (from 5 to 85% acetonitrile containing 0.1% TFA for 30 min at a flow rate of 1.5 mL/min and UV detection at 230 and 254 nm) with eluent A (0.1% (*v*/*v*) TFA in water) and eluent B (0.1% (*v*/*v*) TFA in acetonitrile). The appropriate fractions were pooled and lyophilized. Analytical HPLC (Waters 2695, Waters Corporation, Milford, MA, USA) equipped with a SunfireTM C18 column (3.5 μm particle size; 100 mm× 4.6 mm, Waters Corporation, Milford, MA, USA) produced a single peak (t*_R_* 7.29 min, 46.6% AcCN) with at least 98% of the total peak integrals (Figure S5). The solvent system used was the same as that for the semi-preparative HPLC. High-resolution mass spectra (HRMS) recorded on a Thermo Scientific Linear Trap Quadropole (LTQ) Orbitrap Velo mass spectrometer (Thermo Fisher Scientific, Waltham, MA, USA) agreed with the expected mass. [calcd: 3916.37; found: [M + H]^+^ 3916.94] (Figure S6).

### 4.2. Amyloid Fibril Formation

The lyophilized peptide analogue was dissolved in 1,1,1,3,3,3-Hexafluoro-2-propanol (HFIP, Sigma-Aldrich, St Louis, MO, USA) at a concentration of 1 mg/mL, to disassemble preformed aggregates. The solution was divided into aliquots, which were left to dry overnight in a fume hood, at room temperature, until thin peptide-containing films were created. The peptide-containing films were stored at −20 °C. Before use, each peptide-containing film was left at room temperature for 30 min. The films were dissolved in three different buffer solutions: (a) 0.1 mol/L MES buffer (pH = 5.6, Sigma-Aldrich, St Louis, MO, USA), (b) 0.1 mol/L HEPES buffer (pH = 7.4, Sigma-Aldrich, St Louis, MO, USA), (c) 0.1 mol/L Tris buffer (pH = 8.4, Sigma-Aldrich, St Louis, MO, USA) at a concentration of 20 µmol/L and in distilled water (pH = 5.75) at a concentration of 2.5 mmol/L. After an incubation period of two to three weeks at 37 °C, all peptide solutions were found to form amyloid-like fibrils.

### 4.3. Negative Staining and Transmission Electron Microscopy (TEM)

A total 10 µL of the 20 µmol/L AtPNP-A_36–69_ fibril-containing solutions were applied to 400-mesh glow-discharged and carbon-coated copper TEM grids for 20 min. The excess sample was blotted away with filter paper. Directly afterwards, the grids were stained with a drop of aqueous uranyl acetate 2% (*w*/*v*) for 50 s, and were washed by contact with three drops dH_2_O, before being blotted with a filter paper [75]. The grids were left for a few seconds to air-dry and later examined with a Morgagni^TM^ 268 transmission electron microscope, operated at 80 kV. Digital acquisitions were performed with an 11 Mpixel side-mounted Morada CCD camera (Soft Imaging System, Muenster, Germany).

### 4.4. X-ray Fiber Diffraction

A total 2.5 mol/L of AtPNP-A_36–69_ aqueous solution was incubated for two weeks, to form viscous solutions, which facilitate the formation of oriented fibers. A droplet (5 μL) of the AtPNP-A_36–69_ fibril-containing solution was placed between aligned glass capillaries with wax-covered ends, spaced 2 mm apart and mounted horizontally on a glass slide, as collinearly as possible. The sample was left to air dry at ambient temperature and humidity, for 30–60 min in order to form an oriented fiber, suitable for X-ray fiber diffraction [76]. The diffraction pattern was collected using a SuperNova-Agilent Technologies X-ray generator equipped with a 135 mm ATLAS CCD detector and a 4-circle kappa goniometer, at the Institute of Biology, Medicinal Chemistry and Biotechnology, National Hellenic Research Foundation (CuKa high-intensity X-ray micro-focus source, k 51.5418 Å), operated at 50 kV, 0.8 mA. The oriented fiber sample was mounted onto the goniometer. The specimen-to-film distance was set at 52 mm, whereas exposure time was set to 400 s [77]. The X-ray patterns were initially viewed using the program CrysAlisPro [78] and subsequently displayed and measured with the aid of the program iMosFLM [79].

### 4.5. Attenuated Total Reflectance Fourier-Transform Infrared Spectroscopy (ATR FT-IR) Spectroscopy

A total 5 µL of the 2.5 mmol/L AtPNP-A_36–69_ aqueous solution was applied on a front-coated mirror plate (SpectRIM, Tienta Sciences, Inc. Indianapolis, IN, USA) and was left to air-dry slowly at ambient conditions, in order to form a thin hydrated peptide-containing film. IR spectra were obtained from this film at a resolution of 4 cm^−^^1^, using an IR microscope (IRScope II, BrukerOPTICS, Bruker Optik GmbH, Ettlingen, Germany), equipped with a Ge ATR objective lens (20×) and attached to an FT spectrometer (Equinox 55, BrukerOPTICS, Bruker Optik GmbH, Ettlingen, Germany). In total, ten spectra, 32-scans each, were collected from each of the samples and averaged to improve the Sound/Noise (S/N) ratio [80]. All spectra are shown in the absorption mode after correction for the wavelength dependence of the penetration depth (dp analogous to k). Absorption band maxima were determined from the minima in the second derivative of the corresponding spectra. Derivatives were computed analytically using routines of the Bruker OPUS/OS2 software (Bruker Optik GmbH, Ettlingen, Germany), including smoothing over a 12 cm^−1^ range around each data point, performed by the Savitsky-Golay algorithm [81]. Smoothing over narrower ranges resulted in deterioration of the S/N ratio and at the same time did not increase the number of minima that was observed that could be identified with certainty. The minima in the second derivative were used to determine the corresponding absorption band maxima [77]. Data was visualized using OriginPro 7 (OriginLab Corporation, Northampton, MA, USA).

### 4.6. Thioflavin T (ThT) Kinetic Assay

ThT fluorescence measurements were conducted at 37 °C, in black 96-well plates with flat, clear bottoms, using a Tecan Spark microplate reader. The top of the plates was sealed with microplate covers and the fluorescence readings were performed through the bottom. A 444 nm filter was used for excitation and a 484 nm filter for emission. HFIP peptide films were dissolved in dimethylsulphoxide (DMSO) and diluted in the three buffers for a final DMSO concentration of less than 5% (*v*/*v*). The reaction solutions contained freshly prepared 20 μmol/L soluble AtPNP-A_36–69_ solutions and 50 μmol/L ThT (Sigma^©^) in dH_2_O and had a final volume of 100 µL (9 peptide-analogue: 1 ThT). ThT fluorescence was also measured in the dH_2_O as background. Each experiment was conducted in triplicates. Measurement lasted for 50 h and fluorescence readings were collected every 15 min. Before each measurement, the plate was agitated for 10 s at 270 rpm. ThT background fluorescence was subtracted from the sample readings at each time point. Standard deviation was calculated, and the data was normalized on a scale of 0 to 100 arbitrary units. Data was analyzed and visualized using RStudio (packages ggpubr, rstatix, and ggplot2).

### 4.7. Congo Red Birefringence Assay

A total 5 μL of the 20 µmol/L AtPNP-A_36–69_ solutions were placed on glass slides and left to air-dry at ambient temperature and humidity for a few minutes until a peptide film on top of the slide plate was produced. The films containing amyloid-like fibrils were stained with a 10 mmol/L Congo Red (Sigma^©^) solution in PBS (137 mmol/L NaCl, 27 mmol/L KCl, 100 mmol/L Na_2_HPO_4_, 18 mmol/L KH_2_PO_4_, pH = 7.4) or a 1% (*w*/*v*) Congo Red solution in dH_2_O (pH 5.75) for approximately 30 min [82,83,84]. Excess stain was removed by several washes with either 90% ethanol or tap water and were left to dry approximately for 10 min. The samples were observed under bright field illumination and between crossed polars, using a Leica MZ7.5 polarizing stereomicroscope (Leica Camera AG, Weltzar, Germany), equipped with a Sony *α*6000 camera (Sony, Tokyo, Japan).

### 4.8. Protein-Protein Interaction Network

A protein-protein interaction (PPI) network for AtPNP was created using stringApp [85], an application that allows the direct import of networks from STRING [57], a public database of known and predicted PPIs, into Cytoscape [58]. A cutoff interaction score of 0.7 was selected representing partners of high confidence [86]. To increase the amount of valuable information we get, the network was expanded with 20 additional interactors with a selectivity cutoff of 0.7 and was further studied by performing an enrichment analysis, setting the enrichment significance threshold at 0.05. The resulted network consisted of 32 proteins and 85 PPIs. The experimental association of the proteins in the *Arabidopsis thaliana* network with amyloid formation was detected through an extensive literature search.

## 5. Conclusions

In summary, our data suggest that AtPNP_36–69_ possesses characteristic amyloidogenic properties. Higher pH values induce the formation of robust and well-defined AtPNP-A_36–69_ amyloid fibrils, unlike most mammalian amyloidogenic proteins, which form amorphous aggregates at corresponding pH values. This statement hints at the possibility that plant protein amyloid formation is affected differently by environmental conditions than that of mammalian proteins. Furthermore, our computational work provides insight into the potential role of amyloid formation in plants’ defense mechanisms. Nevertheless, several questions still remain unanswered. Further experimental studies on the nature of plant proteins which tend to self-assemble into amyloids should help elucidate the in vivo contribution of such ultrastructures into plant physiology.

## Figures and Tables

**Table 1:** Bands observed in the ATR FT-IR spectra obtained from thin films, containing suspensions of fibrils, produced by the AtPNP-A_36–69_ and their tentative assignments. TFA: trifluoracetic acid.

Band (cm^−1^)	Assignment
1173	TFA
1203	TFA
1234	β-sheet (amide III)
1404	Asp
1443	Pro (CH2 bend)
1516	Tyr
1533	β-sheet (amide II)
1552	β-sheet (amide II)
1633	β-sheet (amide I)
1643	β-sheet (amide I)
1666	β turn

## Data Availability

Data available within the article or Supplementary Materials.

## Supplementary Materials

The following are available online at https://www.mdpi.com/article/10.3390/plants11010009/s1, Figure S1. Sequence alignment of hANP and AtPNP-A [29,87,88]. Figure S2. Multiple sequence alignment of all plant natriuretic peptides. Each line represents an entry, obtained from the protein database UniProt [88,89,90]. Figure S3. Secondary structure prediction of AtPNP-A from the SecStr algorithm [62] and PORTER [61]. Figure S4. Prediction of AMYLPRED [91] algorithm for Antimicrobial peptide 2 (P86706) of Cocos nucifera, Defensin-like protein 1 (P69241) and Defensin-like protein 2 (P30230) of Raphanus sativus, Defensin-like protein 16, Hevein-like preprotein (P43082) and AtPNP-A (Q9ZV52) of Arabidopsis thaliana and Pro-hevein (P02877) of Hevea brasiliensis. Figure S5. Analytical HPLC chromatogram of peptide-analogue AtPNP-A36-69. Figure S6. Mass spectra of peptide-analogue AtPNP-A36-69.

## Abbreviations

NPs, Natriuretic Peptides; PNPs, Plant NPs; ANP, Atrial Natriuretic Peptide; hANP, human ANP; BNP Brain Natriuretic Peptide; AtPNP-A, *Arabidobsis thaliana* plant natriuretic peptide-A; TFA, Trifluoroacetic Acid; TEM, Transmission Electron Microscopy; IAA, Isolated Atrial Amyloidosis; PR1, Pathogenesis Related Protein-1; PDF1.2, Defensin-like protein 16; PR4, hevein-like pre-pro-protein; PDB, Protein Data Bank; Fmoc, 9-Fluorenylmethoxycarbonyl; DIC, Diisopropylcarbodiimide; HOBt, Hydroxybenzotriazol; DMF, Dimethylformamide; But, tert-Butyl; Boc, 2,2,4,6,7-pentamethyldihydrobenzofuran-5-sulfonyl; tert-butyloxy-carbonyl; HPLC, High-Performance Liquid Chromatography; HRMS, High Resolution Mass Spectra; HFIP, 1,1,1,3,3,3-Hexafluoro-2-Propanol; DMSO, Dimethylsulphoxide; ATR FT-IR, Attenuated Total Reflectance Fourier-Transform Infrared; Linear Trap Quadropole (LTQ).
