# Supplementary material for: Arabidopsis thaliana Plant Natriuretic Peptide Active Domain Forms Amyloid-like Fibrils in a pH-Dependent Manner
Source: Plants (Basel). 2021 Dec 21;11(1):9. doi: 10.3390/plants11010009 (PMC8747288; doi:10.3390/plants11010009)
Supplement: Supplementary file 1 [file plants-11-00009-s001.zip › Supplementary_File_1.pdf]

# *Arabidopsis thaliana* plant natriuretic peptide active domain forms amyloid-like fibrils in a pH-dependent manner

Georgia I. Nasi[1,†], Foteini D. Aktypi[1,†], Panagiotis M. Spatharas[1,‡], Nikolaos N. Louros[1,§,‖], Paraskevi L. Tsiolaki[1,¶], Vassiliki Magafa[2], Ioannis P. Trougakos[1] and Vassiliki A. Iconomidou[1,*]

[1] Section of Cell Biology and Biophysics, Department of Biology, School of Sciences, National and Kapodistrian University of Athens, Panepistimiopolis, 157 01 Athens, Greece
[2] Department of Pharmacy, University of Patras, 265 04 Patras, Greece

[*] Correspondence: veconom@biol.uoa.gr; Tel.: +30-210-7274871; Fax: +30-210-7274254

[†] These authors contributed equally to this work.

[‡] Present Address: European Molecular Biology Laboratory, Hamburg Unit, Notkestrasse 85, 22607 Hamburg, Germany.
[§] Present Address: KU Leuven, Department of Cellular and Molecular Medicine, VIB-KU Leuven, 3000 Leuven, Belgium.
[‖] Present Address: VIB Center for Brain and Disease Research, Switch Laboratory, Herestraat 49, 3000 Leuven, Belgium.
[¶] Present Address: Department of Biochemistry and Biophysics, University of California, San Francisco, CA 94143, USA.

Supplementary File 1

# Sequence alignments

## Sequence alignment of hANP and AtPNP-A

The amino acid sequences of the AtPNP-A (Q9ZV52) and the hANP (P01160) were extracted from UniprotKB, free from their signal peptide or pro-peptide sequences. The two peptide sequences were then aligned applying the Needleman-Wunsch algorithm [87] and the result was manually edited utilizing Jalview [88].

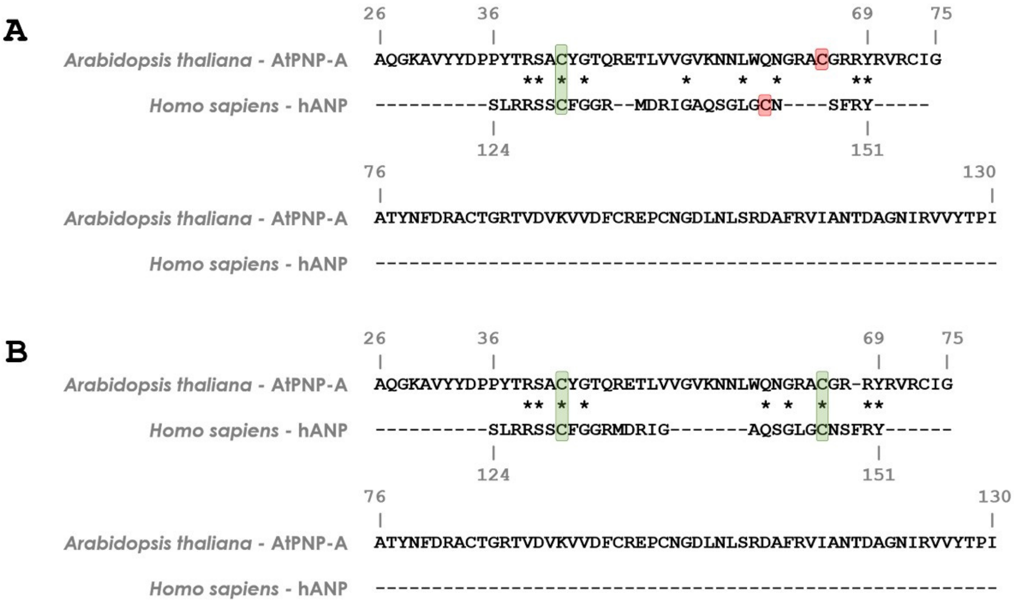


**Supplementary Figure S1. Sequence alignment of hANP and AtPNP-A.** (A) The amino acid sequence homology between hANP and AtPNP-A as suggested after the molecular identification of the AtPNP-A [29]. Green box: aligned cysteines, Red box: not aligned cysteines (B) The alignment of hANP
and AtPNP-A we propose based on the available experimental data supporting the importance of the disulfide bond to obtain their functional structure (green boxes). Asterisks (*) indicate positions which have a single, fully conserved residue.

**Multiple sequence alignment of all know plant natriuretic peptides (PNPs)**

Amino acid sequences of all known plant natriuretic peptides (PNPs) were extracted from UniprotKB [89], with the following accession numbers: Q9ZV52 (*Arabidopsis thaliana*), Q9M0C2 (*Arabidopsis thaliana* ), Q9ZP41 (*Citrus jambhiri*), A0A251V4M4 (*Helianthus annuus*), A0A061GQD7 (*Theobroma cacao*), A0A061GQ10 (*Theobroma cacao*), A0A061GAM9 (*Theobroma cacao*), A0A061GQI5 (*Theobroma cacao*), A0A061GQU2 (*Theobroma cacao*), A0A061GNK9 (*Theobroma cacao*), Q8RWA0 (*Erucastrum strigosum*), A0A4Y1QUF7 (*Prunus dulcis*), A0A5B7B5A2 (*Davidia involucrata*), A0A4Y1RVQ8 (*Prunus dulcis*), Q84V62 (*Hedera helix*). Multiple sequence alignment of all PNPs sequences was performed utilizing Clustal Omega [90] and Jalview [88].

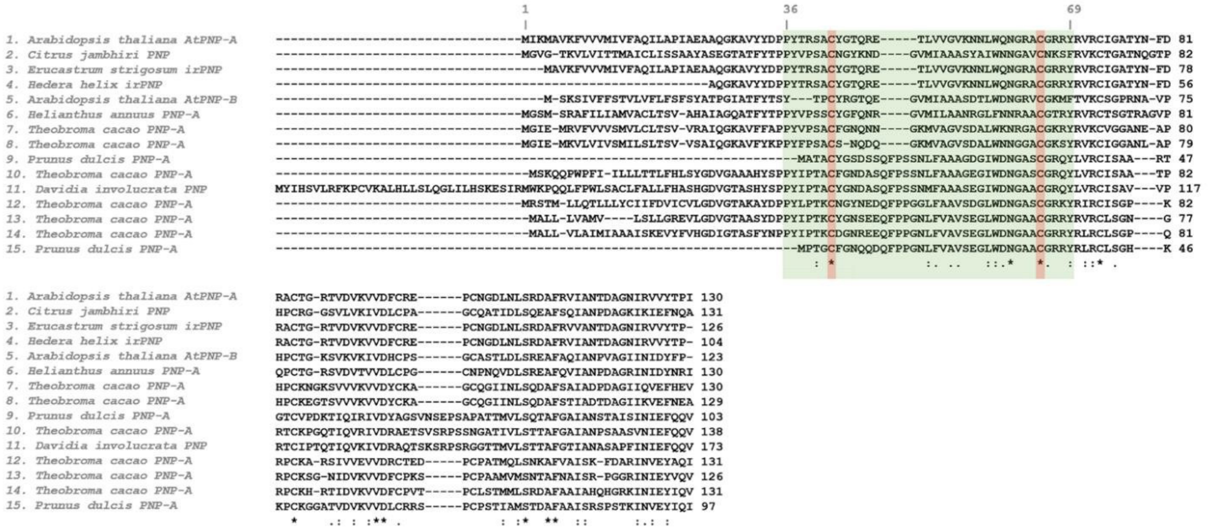


**Supplementary Figure S2. Multiple sequence alignment of all plant natriuretic peptides. Each line represents an entry, obtained from the protein database UniProt** [90]. A green box shows the amino acid residues corresponding to the 34 amino acid active domain of the AtPNP-A sequence and the homologous regions corresponding to the rest of the PNPs. As it is observed, the cysteine residues are conserved in all sequences (orange boxes). The corresponding UniProt Accession Numbers (ACs) are given: 1. Q9ZV52, 2. Q9ZP41, 3. Q8RWA0, 4. Q9M0C2, 5. Q84V62, 6. A0A251V4M4, 7. A0A061GQD7, 8. A0A061GQ10, 9. A0A061GQI5, 10. A0A061GQU2, 11. A0A061GAM9, 12. A0A061GNK9. Asterisks (*) indicate positions which have a single, fully conserved residue. Colon (:) indicates conservation between groups of strongly similar properties (> 50% in the Gonnet PAM 250 matrix). Period (.) indicates conservation between groups of weakly similar properties (≤50% and >0 in the Gonnet PAM 250 matrix) [90].
