# Supplementary material for: Arabidopsis thaliana Plant Natriuretic Peptide Active Domain Forms Amyloid-like Fibrils in a pH-Dependent Manner
Source: Plants (Basel). 2021 Dec 21;11(1):9. doi: 10.3390/plants11010009 (PMC8747288; doi:10.3390/plants11010009)
Supplement: Supplementary file 1 [file plants-11-00009-s001.zip › Supplementary_File_2.pdf]

Arabidopsis thaliana plant natriuretic peptide active domain forms amyloid-like fibrils in a pH-dependent manner

Georgia I. Nasi[1,†], Foteini D. Aktypi[1,†], Panagiotis M. Spatharas[1,‡], Nikolaos N. Louros[1,§,‖], Paraskevi L. Tsiolaki[1,¶], Vassiliki Magafa[2], Ioannis P. Trougakos[1] and Vassiliki A. Iconomidou[1,*]

[1] Section of Cell Biology and Biophysics, Department of Biology, School of Sciences, National and Kapodistrian University of Athens, Panepistimiopolis, 157 01 Athens, Greece
[2] Department of Pharmacy, University of Patras, 265 04 Patras, Greece

[*] Correspondence: veconom@biol.uoa.gr; Tel.: +30-210-7274871; Fax: +30-210-7274254

[†] These authors contributed equally to this work.
[‡] Present Address: European Molecular Biology Laboratory, Hamburg Unit, Notkestrasse 85, 22607 Hamburg, Germany.
[§] Present Address: KU Leuven, Department of Cellular and Molecular Medicine, VIB-KU Leuven, 3000 Leuven, Belgium.
[‖] Present Address: VIB Center for Brain and Disease Research, Switch Laboratory, Herestraat 49, 3000 Leuven, Belgium.
[¶] Present Address: Department of Biochemistry and Biophysics, University of California, San Francisco,
CA 94143, USA.

Supplementary File 2

Secondary Structure Analysis

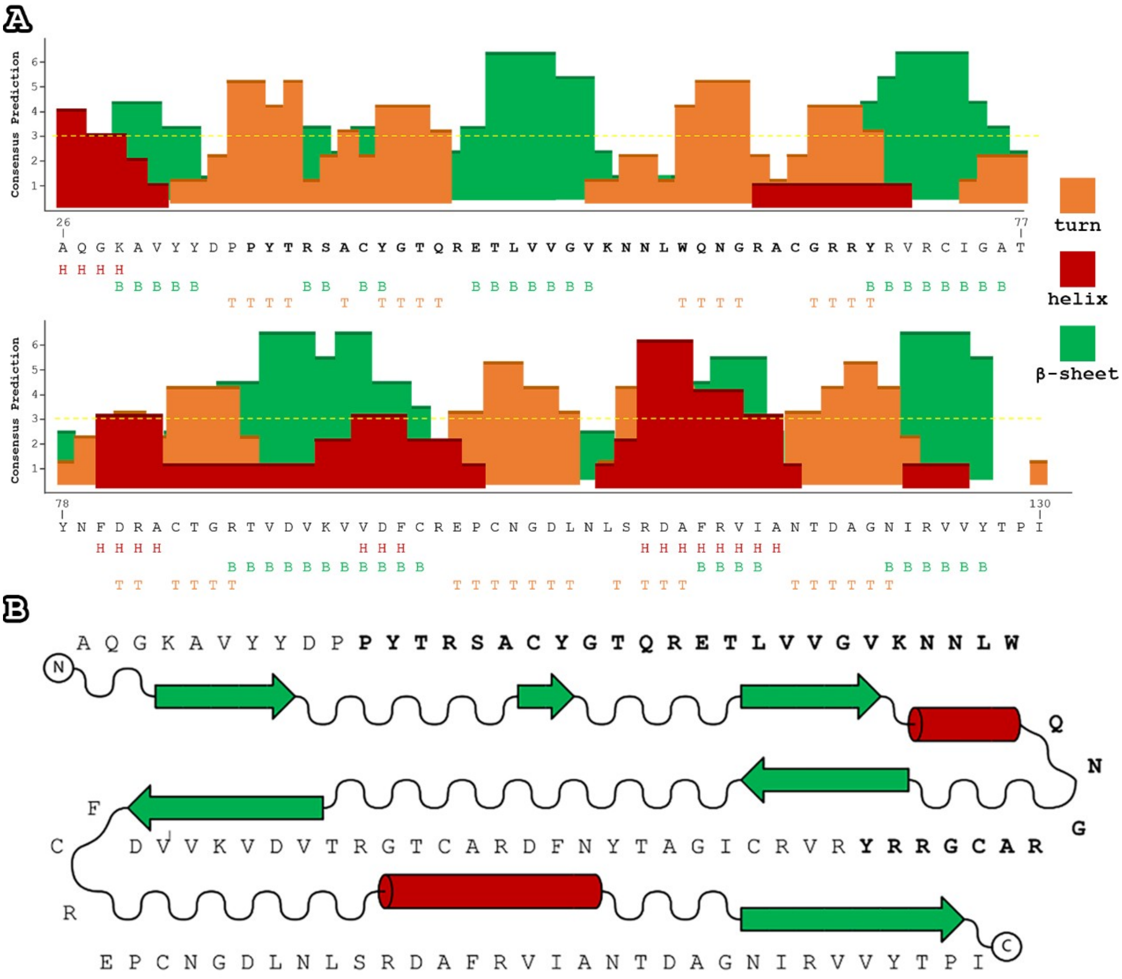

Supplementary Figure S **3. Secondary structure prediction of AtPNP-A from the SecStr algorithm**

[62] **and PORTER** [61]. (A) Secondary structure prediction of AtPNP-A from the SecStr algorithm. The graphical chart represents the secondary structure prediction profile of AtPNP-A per residue (orange: turn, red: helix, green: β-strand). Yellow line illustrates a 'consensus threshold', where at least three (3) out of six (6) methods concur in secondary structure prediction. Bold letters depict the AtPNP-A36-69 peptide-analogue, tested experimentally. B-strands are the main secondary structure element of the AtPNP-A sequence, but there are regions that are predicted as "chameleon sequences" since, according to SECSTR prediction, they tend to adopt a helical or an extended conformation (H : α-helix, B: β-strand, T: β-turn). (B) Secondary structure prediction of AtPNP-A from the PORTER algorithm. According to the prediction, $AtPNP_{36\text{-}69}$ adopts a turn – β-strand – turn – β-strand – helix – turn fold.

```
                 1         10
Antimicrobial    |--------|-
  peptide 2      TESYFVFSVGM
                   *******
------------------------------------------------------------------------------------
                 1        10        20        30        40        50
Defensin like    |--------|---------|---------|---------|---------|-
  protein 1      QKLCERPSGTWSGVCGNNNACKNQCINLEKARHGSCNYVFPAHKCICYFPC
                                                  ********  ******
------------------------------------------------------------------------------------
                 1        10        20        30        40        50
Defensin like    |--------|---------|---------|---------|---------|-
  protein 2      QKLCQRPSGTWSGVCGNNNACKNQCIRLEKARHGSCNYVFPAHKCICYFPC
                                                   ********  ******
------------------------------------------------------------------------------------
                 1        10        20        30        40        50
Defensin like    |--------|---------|---------|---------|---------|-
  protein 16     QKLCEKPSGTWSGVCGNSNACKNQCINLEGAKHGSCNYVFPAHKCICYVPC
                                                  ********  ******
------------------------------------------------------------------------------------
                 1        10        20        30        40        50
                 |--------|---------|---------|---------|---------|
                 AQGKAVYYDPPYTRSACYGTQRETLVVGVKNNLWQNGRACGRRYRVRCIG
                                         ******              *******
  AtPNP-A        51       60        70        80        90        100  105
                 |--------|---------|---------|---------|---------|----|
                 ATYNFDRACTGRTVDVKVVDFCREPCNGDLNLSRDAFRVIANTDAGNIRVVYTPI
                 *            ********               *****       *****
------------------------------------------------------------------------------------
                 1        10        20        30        40        50        60
                 |--------|---------|---------|---------|---------|---------|
                 QQCGRQGGGRTCPGNICCSQYGYCGTTADYCSPTNNCQSNCWGSGPSGPGESASNVRATY
                                                                           ***
                 61       70        80        90        100       110       120
                 |--------|---------|---------|---------|---------|---------|
Hevein-like      HFYNPAQNNWDLRAVSAYCSTWDADKPYAWRSKYGWTAFCGPAGPRGQASCGKCLRVKNT
preproprotein    ****                             ******
                 121      130       140       150       160       170       180
                 |--------|---------|---------|---------|---------|---------|
                 RTNAAVTVRIVDQCSNGGLDLDVAMFNQIDTDGFGYQQGHLIVDYQFVDCGNELIGQPDS
                      ******          ********           *******
                 181      190
                 |--------|-
                 RNMLVSAIDRV
                   ******
------------------------------------------------------------------------------------
                 1        10        20        30        40        50        60
                 |--------|---------|---------|---------|---------|---------|
                 EQCGRQAGGKLCPNNLCCSQWGWCGSTDEYCSPDHNCQSNCKDSGEGVGGGSASNVLATY
                                ********                                 *****
                 61       70        80        90        100       110       120
                 |--------|---------|---------|---------|---------|---------|
  Pro-hevein     HLYNSQDHGWDLNAASAYCSTWDANKPYSWRSKYGWTAFCGPVGAHGQSSCGKCLSVTNT
                 ****                             ******
                 121      130       140       150       160       170       180
                 |--------|---------|---------|---------|---------|---------|
                 GTGAKTTVRIVDQCSNGGLDLDVNVFRQLDTDGKGYERGHITVNYQFVDCGDSFNPLFSV
                        ****           ******              ********        *****
                 181
                 |------
                 MKSSVIN
```

Supplementary Figure S4. Prediction of AMYLPRED [91] algorithm for Antimicrobial peptide 2 (P86706) of *Cocos nucifera*, Defensin-like protein 1 (P69241) and Defensin-like protein 2
(P30230)

of *Raphanus sativus*, Defensin-like protein 16, Hevein-like preprotein (P43082) and AtPNP-A (Q9ZV52) of *Arabidopsis thaliana* and Pro-hevein (P02877) of *Hevea brasiliensis*. Predicted "aggregation-prone" segments by AMYLPRED are shown in green and are marked with '*' under the sequence. The amyloidogenic profiles of Defensin-like protein 1, Defensin-like protein 2 and Defensin-like protein 16 are the same, implying that Defensin-like protein 16 has the ability to form amyloid fibrils. Additionally, the amyloidogenic profiles of Hevein-like preprotein and Pro-hevein are similar, suggesting that Hevein-like preprotein can form amyloid fibrils. Aggregation-prone regions are also predicted in AtPNP-A, with one of them located in the 36-69 region.
