# Supplementary material for: Arabidopsis thaliana Plant Natriuretic Peptide Active Domain Forms Amyloid-like Fibrils in a pH-Dependent Manner
Source: Plants (Basel). 2021 Dec 21;11(1):9. doi: 10.3390/plants11010009 (PMC8747288; doi:10.3390/plants11010009)
Supplement: Supplementary file 1 [file plants-11-00009-s001.zip › Supplementary_File_3.pdf]

Arabidopsis thaliana plant natriuretic peptide active domain forms amyloid-like fibrils in a pH-dependent manner

Georgia I. Nasi[1,†], Foteini D. Aktypi[1,†], Panagiotis M. Spatharas[1,‡], Nikolaos N. Louros[1,§,‖], Paraskevi L. Tsiolaki[1,¶], Vassiliki Magafa[2], Ioannis P. Trougakos[1] and Vassiliki A. Iconomidou[1,*]

[1] Section of Cell Biology and Biophysics, Department of Biology, School of Sciences, National and Kapodistrian University of Athens, Panepistimiopolis, 157 01 Athens, Greece
[2] Department of Pharmacy, University of Patras, 265 04 Patras, Greece

[*] Correspondence: veconom@biol.uoa.gr; Tel.: +30-210-7274871; Fax: +30-210-7274254

[†] These authors contributed equally to this work.
[‡] Present Address: European Molecular Biology Laboratory, Hamburg Unit, Notkestrasse 85, 22607 Hamburg, Germany.
[§] Present Address: KU Leuven, Department of Cellular and Molecular Medicine, VIB-KU Leuven, 3000 Leuven, Belgium.
[‖] Present Address: VIB Center for Brain and Disease Research, Switch Laboratory, Herestraat 49, 3000 Leuven, Belgium.
[¶] Present Address: Department of Biochemistry and Biophysics, University of California, San Francisco, CA 94143, USA.

Supplementary File 3

Purification and Analysis by RP-HPLC

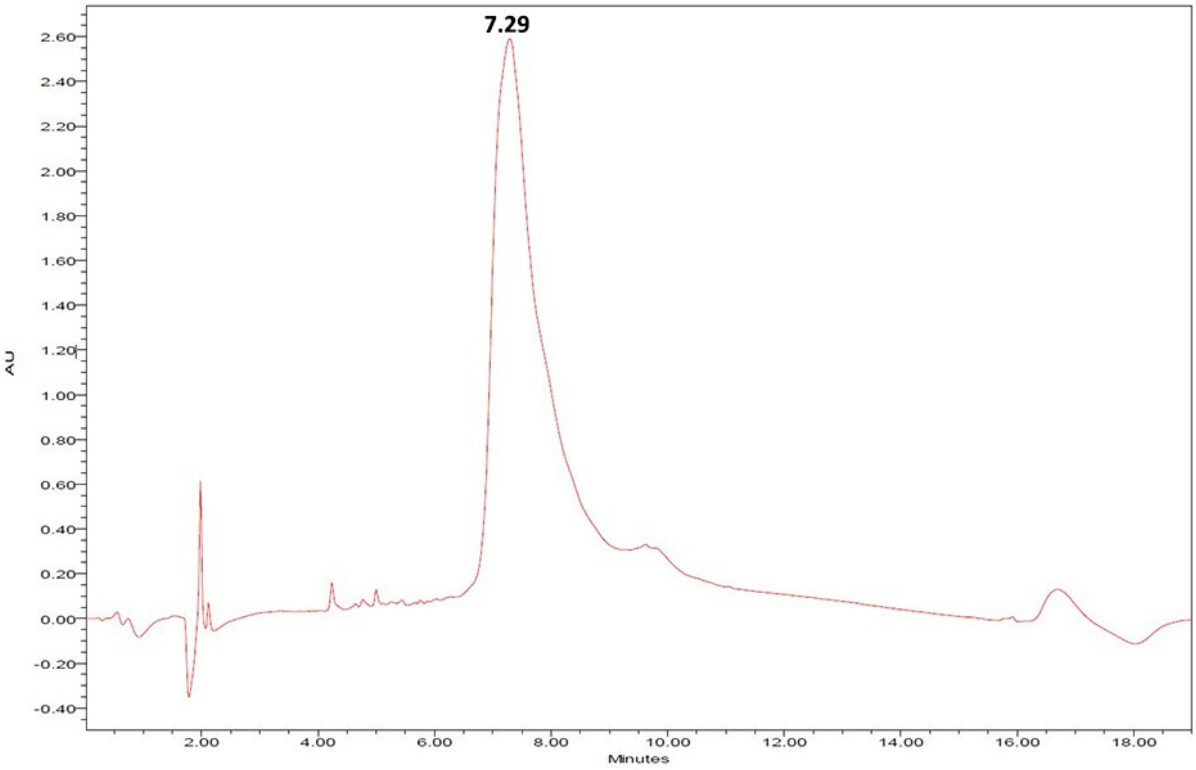

Supplementary Figure S5. Analytical HPLC chromatogram of peptide-analogue AtPNP-A$_{36\text{-}69}$. A single peak (t_R 7.29 min, 46.6% AcCN) was produced, with at least 98% of the total peak integrals.

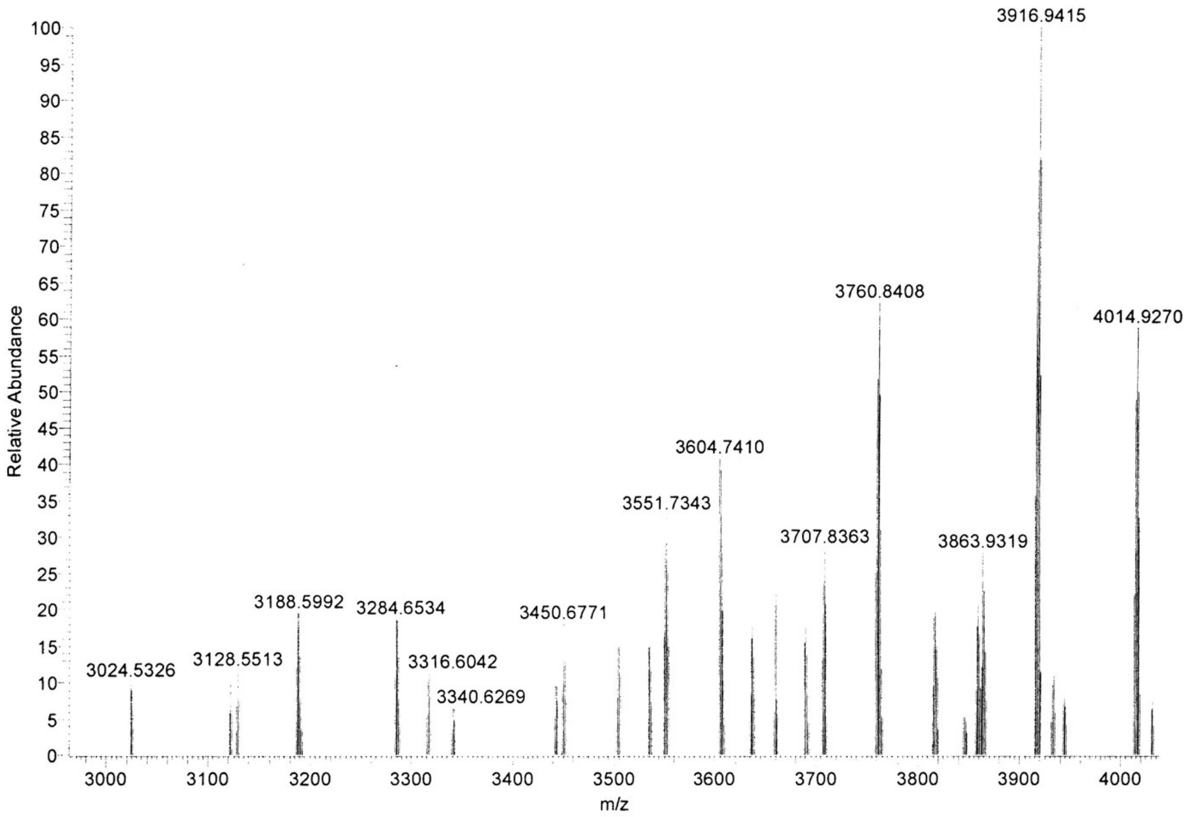

Supplementary Figure S6. Mass spectra of peptide-analogue AtPNP-A$_{36\text{-}69}$. The recorded mass agrees with the expected one [calculated: 3916.37; found: $[M+H]^+$ 3916.94]..
